# A new harmony box supplemented with gonial angle and age based on a growing Swiss population

**DOI:** 10.1007/s00056-024-00569-4

**Published:** 2025-01-15

**Authors:** Nele Lorenz, Despina Koletsi, Raphael Patcas, Rebecca Jungbauer, Vasiliki Koretsi

**Affiliations:** 1 Clinic of Orthodontics and Pediatric Dentistry, Center of Dental Medicine, University of Zurich, Plattenstrasse 11, 8032 Zurich, Switzerland; 2 Meta-Research Innovation Center at Stanford (METRICS), Stanford University, Stanford, CA USA; 3 Department of Orthodontics, University Hospital Regensburg, Regensburg, Germany

**Keywords:** Orthodontic treatment planning, Individualized cephalometric measurements, Floating norms, Craniofacial growth study, Harmony box, Planung kieferorthopädischer Behandlungen, Individualisierte kephalometrische Messungen, Veränderliche Normen, Studie zum kraniofazialen Wachstum, Harmoniebox

## Abstract

**Purpose:**

The scope of the present study was to create a new harmony box by adding two diagnostically and clinically important cephalometric variables, the gonial and interincisal angles, while also considering the effect of sex and age for a growing Swiss population.

**Methods:**

A healthy sample with an overjet and overbite between 2 and 4 mm, and 1.5 and 4.5 mm, respectively, of the Zurich Craniofacial Growth Study was considered. Pairwise correlations between the cephalometric angles were evaluated with the Pearson correlation coefficient (r). Regression models were built for each cephalometric variable serving as the dependent one. The Akaike Information Criterion and the Bayesian Information Criterion were used to structure and select the final multivariable regression model. Bland–Altman plots and the Lin’s concordance correlation coefficient were used to assess inter- and intraexaminer agreement.

**Results:**

The sample consisted of 301 individuals with a mean age of 13.4 years. Measurement concordance was confirmed both within and between examiners. The strongest correlations were observed between the angles SNB and SNA (r = 0.81), ArGoMe and SN-ML (r = 0.57), and SN-ML and SNB (r = 0.56). The SNB angle qualified as the dependent variable in the multivariable regression that framed the newly provided harmony box, with the predictor variables age (*p* < 0.001) and the angles SNA (*p* < 0.001), SN-ML (*p* < 0.001), SN-NL (*p* = 0.005), NSBa (*p* = 0.001), and ArGoMe (*p* < 0.001). The interincisal angle did not increase the robustness of the model and was excluded (*p* > 0.05).

**Conclusion:**

Contrary to the interincisal angle, gonial angle and age qualified for inclusion in the new harmony box for individualized cephalometrics in a sample of healthy schoolchildren from Zurich, Switzerland.

**Supplementary Information:**

The online version of this article (10.1007/s00056-024-00569-4) contains supplementary information, which is available to authorized users.

## Abstract (de)

**Zielsetzung:**

Ziel der vorliegenden Studie war es, eine neue Harmoniebox zu erstellen, indem 2 diagnostisch und klinisch wichtige kephalometrische Variablen, der Gonion- und der Interinzisalwinkel, hinzugefügt wurden, und dabei auch die Auswirkungen von Geschlecht und Alter für eine sich im Wachstum befindliche Schweizer Stichprobe zu berücksichtigen.

**Methoden:**

Für die Studie wurde eine Stichprobe gesunder Personen mit einem Overjet und einem Overbite zwischen 2 und 4 bzw. 1,5 und 4,5 mm aus der Zürcher Wachstumsstudie herangezogen. Die paarweisen Korrelationen zwischen den kephalometrischen Winkeln wurden mit dem Pearson-Korrelationskoeffizienten (r) ausgewertet. Für jede kephalometrische Variable, die als abhängige Variable diente, wurden Regressionsmodelle erstellt. Das Akaike-Informationskriterium und das Bayes-Informationskriterium wurden verwendet, um das endgültige multivariable Regressionsmodell zu strukturieren und auszuwählen. Bland-Altman-Diagramme und der Konkordanz-Korrelationskoeffizient nach Lin wurden zur Bewertung der Inter- und Intrauntersucher-Übereinstimmung eingesetzt.

**Ergebnisse:**

Die Stichprobe bestand aus 301 Probanden mit einem Durchschnittsalter von 13,4 Jahren. Die Konkordanz der Messungen wurde sowohl bei den einzelnen Untersuchern als auch zwischen den Untersuchern bestätigt. Die stärksten Korrelationen wurden zwischen den Winkeln SNB und SNA (r = 0,81), ArGoMe und SN-ML (r = 0,57) sowie SN-ML und SNB (r = 0,56) beobachtet. Der SNB-Winkel wurde als abhängige Variable in der multivariablen Regression definiert, aus der die neu erstellte Harmonie-Box entstand, mit den Prädiktorvariablen Alter (*p* < 0,001) und den Winkeln SNA (*p* < 0,001), SN-ML (*p* < 0,001), SN-NL (*p* = 0,005), NSBa (*p* = 0,001) und ArGoMe (*p* < 0,001). Der Interinzisalwinkel erhöhte die Robustheit des Modells nicht und wurde ausgeschlossen (*p* > 0,05).

**Schlussfolgerung:**

Im Gegensatz zum Interinzisalwinkel waren der Gonialwinkel und das Alter für die Aufnahme in die neue Harmoniebox für die individualisierte Kephalometrie bei einer Stichprobe gesunder Schulkinder aus Zürich, Schweiz, geeignet.

## Introduction

Following the introduction of the new standardized lateral radiographic technique and the associated need of a metric analysis in 1931 [1, 2], lateral cephalometric radiographs with the corresponding cephalometric analyses became essential diagnostic and treatment planning tools, being part of the necessary orthodontic set of records in multiple treatment stages and the majority of patients [3, 4]. A plethora of cephalometric analyses have been developed so far [5]. Their scope is to compare each patient’s cephalometric measurements to the respective population norms. That approach might be misleading, since individuals can still present normal occlusions spread across a large range of skeletal configurations [6, 7]. In this context and if it is assumed that cephalometric data fall under a Gaussian distribution, a cephalometric value within the third standard deviation, which would represent 2.1% of the population on each side of the mean, might still be considered normal for a given individual [8, 9].

On the other hand, a cephalometric value lying within the first standard deviation and representing 34.1% of the population on each side of the mean might be considered abnormal, if all other components constituting a malocclusion fall considerably apart [3]. The fact that both aforementioned scenarios may be plausible can be attributed to the complex, multifactorial, and interdependent nature of growth and malocclusions. Enlow and Hans [10] developed the counterpart principle of craniofacial growth, according to which any given part of the face or cranium specifically links to other facial and cranial counterparts via regional relationships. Furthermore, Solow [11] introduced the concept of craniofacial pattern, instead of considering isolated cephalometric variables. This was done because significant associations between anteroposterior and vertical cephalometric variables were identified. The advantage of the approaches utilizing the relationships among different craniofacial parts or variables lies on the individualized, floating, norms: the ideal value of the cephalometric variable under consideration is expressed as a function of the values of the patient’s other cephalometric variables.

The first effort to put together cephalometric variables with synergistic interactions was made by Hasund et al. [12] with the Bergen box. The Bergen box has evolved to the current harmony box [13–15], which is based on the intercorrelations instead of merely on the population means among the included cephalometric variables. It is considered a valuable diagnostic tool nowadays. Briefly, the cephalometric angles SNA, SN-NL, NSBa, SN-ML, and SNB are represented in the harmony box, and there is also information on the facial types, as originally proposed by Björk [16]. A skeletal pattern is considered harmonious if the patient’s cephalometric values theoretically lie on a straight line (the line of harmony) or within the tolerated limit of variability across the represented cephalometric angles, i.e., the included anteroposterior and vertical cephalometric variables are in balance with each other. The tolerated limit of variability for each cephalometric variable is calculated based on the remaining cephalometric variables of the box and is the range of standard errors for each patient’s line of harmony. In this context, it is possible to find harmonious combinations of the cephalometric variables represented in the harmony box for any patient’s given cephalometric values [14].

Björk’s facial polygon [16] for analyzing the interactive effects of anteroposterior and vertical cephalometric variables contains the gonial angle, among other angular and linear measurements. Furthermore, the shape of the lower mandibular border belongs to Björk’s structural signs of mandibular growth rotation [17]. Although the former does not exclusively refer to the inclination of lower mandibular border [17], the tangent to the lower mandibular border forms an arm for the gonial angle. Moreover, larger gonial angles are typical of hyperdivergent skeletal configurations as compared to hypodivergent ones [18, 19]. In addition to this, morphometric studies consistently identify the gonial area as a source of facial morphological variation, approximately ranging from 9 to 12.5% in orthodontic populations [20–22]. Finally, as far as the incisors are concerned, the interincisal angle is also part of Björk’s structural signs of mandibular growth rotation [17]. Compensated incisors are very common in anteroposterior and vertical malocclusions [23–25] and their positioning is of paramount importance in orthodontics due to their impact on the patient’s smile and the esthetic perception of the profile [26].

In line with the above and given the usefulness of the harmony box in diagnostics and treatment planning, together with the importance of the gonial as well as the interincisal angles in orthodontics, the aim of the present cross-sectional study was to provide a new harmony box supplemented with the gonial and interincisal angles, while considering also the effect of age and sex for a growing Swiss population.

## Materials and methods

The sample for this study comprised the cephalometric radiographs of the Zurich Craniofacial Growth Study conducted from 1981–1984 at the Clinic of Orthodontics and Pediatric Dentistry, University of Zurich, Switzerland. For the present study, an exception to the consent requirement was granted by the Cantonal Ethics Committee Zurich (BASEC-No.: 2022-01857). Additionally, the protocol of the present study was registered online in advance [27].

The Zurich Craniofacial Growth Study consists of cephalometric and hand–wrist radiographs of healthy Caucasian individuals between 6 and 18 years of age. Individuals with systemic diseases, syndromes or under medication were excluded, yet individuals with potential habits, malocclusions, enlarged tonsils, etc., were included. Individuals had no previous orthodontic treatment and were consecutively selected from public schools in Zurich, Switzerland.

The cephalometric radiographs were taken close to each individual’s birthday in centric occlusion. Ear rods into the external auditory canals and a nasal positioner stabilized the head with the Frankfort horizontal plane parallel to the floor. Subsequent cephalometric point identification and positioning was undertaken by three examiners, who had been previously calibrated by a senior orthodontist. A tablet digitizer (NumonicsAccuGrid, Lansdale, PA, USA) with a resolution of 1 milli-inch and a self-written software were then used to digitize points and compute cephalometric measurements (Appendix 1).

For the present study, only a subsample of the original sample was retrospectively considered. Due to the absence of data on molar occlusion, only data on overjet and overbite were considered. Specifically, the present subsample consisted of individuals with an overjet from 2–4 mm and an overbite from 1.5–4.5 mm. Those ranges were chosen to reflect clinically relevant normality, given the fact that overjet and overbite do not remain stable with increasing age, and overbite tends to decrease with increasing age [28].

### Statistical analyses

Data were checked for normality for all cephalometric measurements, statistically through the Shapiro–Wilk test, and visually through Q–Q plots. Descriptive statistics included means, standard deviations (SDs), and minimum and maximum values in normally distributed data. The SNA, SN-NL, NSBa, SN-ML, SNB, ArGoMe, and U1-L1 cephalometric angles were tested for pairwise correlations through the Pearson correlation coefficient (r). For each cephalometric variable serving as the dependent variable, a regression model was built, based on the coefficients. The remaining variables (including age and sex) were inserted in the model sequentially, one at a time (forward stepwise variable selection in order of correlation). To balance model fit and its complexity, two information criteria were assessed to structure and select the final multivariable regression model—first, the Akaike Information Criterion (AIC), and, second, the Bayesian Information Criterion (BIC). The model, which minimized the considered information criteria, was selected for each dependent variable. A final multivariable linear regression model was built, representing the variable serving as an outcome (dependent variable) and the association of variables, contributing to the most robust and stable model based on the regression coefficient (R-squared). In the final model, the SNB angle qualified as the dependent variable.

Bland–Altman plots and the Lin’s concordance correlation coefficient (Lin’s CCC) were used to assess inter- and intraexaminer agreement for all variables in 50 of the included records (approximately 17% of the sample). The level of statistical significance was set at *p* < 0.05. All statistical analyses were conducted with Stata version 15.1 software (Stata Corporation, College Station, TX, USA).

## Results

The sample consisted of 301 individuals with a mean age of 13.4 years (standard deviation [SD] 2.5). There was a balanced male to female ratio with 147 (48.8%) males and 154 females (51.2%). The descriptive statistics for the sample are presented in Table 1.

**Table 1:** Description of the demographic characteristics and variables assessed in the present sample (*n* = 301) Beschreibungen der in der vorliegenden Stichprobe (*n* = 301) bewerteten demographischen Merkmale und Variablen

Variables	Mean (*n*)^a^	SD (%)^a^	Min	Max
Age	13.4	2.5	6.0	18.0
Sex^a^				
*Female*	154	51.2	–	–
*Male*	147	48.8	–	–
SNA	81.5	3.4	70.8	90.9
SNB	78.9	3.1	71.0	88.1
NSBa	130.5	4.6	118.9	144.5
SN-NL	7.5	3.1	−1.1	15.2
SN-ML	33.1	4.2	22.9	45.8
ArGoMe	127.9	5.2	113.5	143.0
U1-L1	131.1	9.0	105.0	160.6
ANB	2.6	2.1	−2.8	10.9
NL-ML	25.6	4.4	11.3	38.2
Overjet	3.1	0.6	2.0	4.0
Overbite	3.0	0.9	1.5	4.5

*SD* standard deviation, *Min* minimum, *Max* maximum

^a^ represented in numbers as absolute (*n*) and relative frequencies (%)

The concordance in measurements for all cephalometric variables was confirmed both within (Lin’s ICC range 0.979–0.994) and between (Lin’s CCC range 0.973–0.995) the examiners. For angular variables, the largest mean intraexaminer difference was 0.10° between measurements, while the largest mean interexaminer difference was 0.39° between measurements (Appendix 2).

Significant correlation coefficients at *p* < 0.001 were observed between the cephalometric angles SNB and SNA, NSBa and SNA, NSBa and SNB, SN-NL and SNA, SN-NL and SNB, SN-NL and NSBa, SN-ML and SNA, SN-ML and SNB, SN-ML and NSBa, SN-ML and SN-NL, and ArGoMe and SN-ML. The strongest correlations were observed between the angles SNB and SNA (r = 0.81), ArGoMe and SN-ML (r = 0.57), and SN-ML and SNB (r = 0.56). The interincisal angle did not have any statistically significant correlations with any of the other cephalometric angles and, therefore, it was not further considered (Table 2).

**Table 2:** Pearson correlation coefficients (r) between the cephalometric variables assessed Pearson-Korrelationskoeffizienten (r) zwischen den bewerteten kephalometrischen Variablen

	SNA	SNB	NSBa	SN-NL	SN-ML	ArGoMe	U1-L1
SNA	1.00	–	–	–	–	–	–
SNB	0.81^a^	1.00	–	–	–	–	–
NSBa	−0.29^a^	−0.39^a^	1.00	–	–	–	–
SN-NL	−0.30^a^	−0.43^a^	0.44^a^	1.00	–	–	–
SN-ML	−0.39^a^	−0.56^a^	0.19^a^	0.31^a^	1.00	–	–
ArGoMe	−0.05	−0.06	−0.06	−0.09	0.57^a^	1.00	–
U1-L1	−0.09	−0.03	−0.08	0.01	−0.09	−0.06	1.00

^a^ significant correlation at *p* < 0.001; no other significant correlations were detected at *p* < 0.05

The SNB angle qualified as the dependent variable to build the structure of the newly provided harmony box, with predictor variables age and the angles SNA, SN-ML, SN-NL, NSBa, and ArGoMe. The multivariable regression model showed that for each degree of increase in the SNA angle, the SNB angle also increased by 0.55° (95% confidence interval [CI]: 0.49, 0.60; *p* < 0.001). For each degree of increase in the SN-ML or SN-NL angles, the SNB angle decreased by 0.26° (95%CI: −0.32, −0.20; *p* < 0.001) and 0.10° (95%CI: −0.16, −0.03; *p* = 0.005), respectively. Furthermore, a rise by one degree in the NSBa angle caused a reduction in the SNB angle by 0.07° (95%CI: −0.11, −0.03; *p* = 0.001). Finally, as the ArGoMe angle increased by 1°, the SNB angle also increased by 0.10° (95%CI: 0.07, 0.16; *p* < 0.001). Individuals’ age was detected as a significant predictor—as individuals became one year older, the SNB angle increased by 0.16° (95%CI: 0.09, 0.23; *p* < 0.001; Table 3).

**Table 3:** Multivariable linear regression representing the relationship of variables contributing to the most robust and stable model^a,b^ Multivariable lineare Regression, Darstellung der Beziehung von Variablen, die zum belastbarsten und stabilsten Modell beitragen^a,b^

Variables^c^	Coefficient	SE	95%CI	*p*-value
SNA	0.55	0.03	0.49, 0.60	< 0.001
SN-ML	−0.26	0.03	−0.32, −0.20	< 0.001
SN-NL	−0.10	0.03	−0.16, −0.03	0.005
NSBa	−0.07	0.02	−0.11, −0.03	0.001
ArGoMe	0.11	0.02	0.07, 0.16	< 0.001
Age	0.16	0.04	0.09, 0.23	< 0.001
Constant	36.73	4.57	27.74, 45.72	< 0.001

*SE* standard error, *CI* confidence interval

^a^ based on Akaike (AIC) and Bayesian (BIC) information criteria (overall R^2^ = 0.78); if similar AIC were observed, the inclusion was based on BIC

^b^ the SNB angle qualified as the dependent variable

^c^ variables inserted sequentially in order of correlation according to Table 2; sex and age were also tested and included, if relevant

**Fig. 1:**
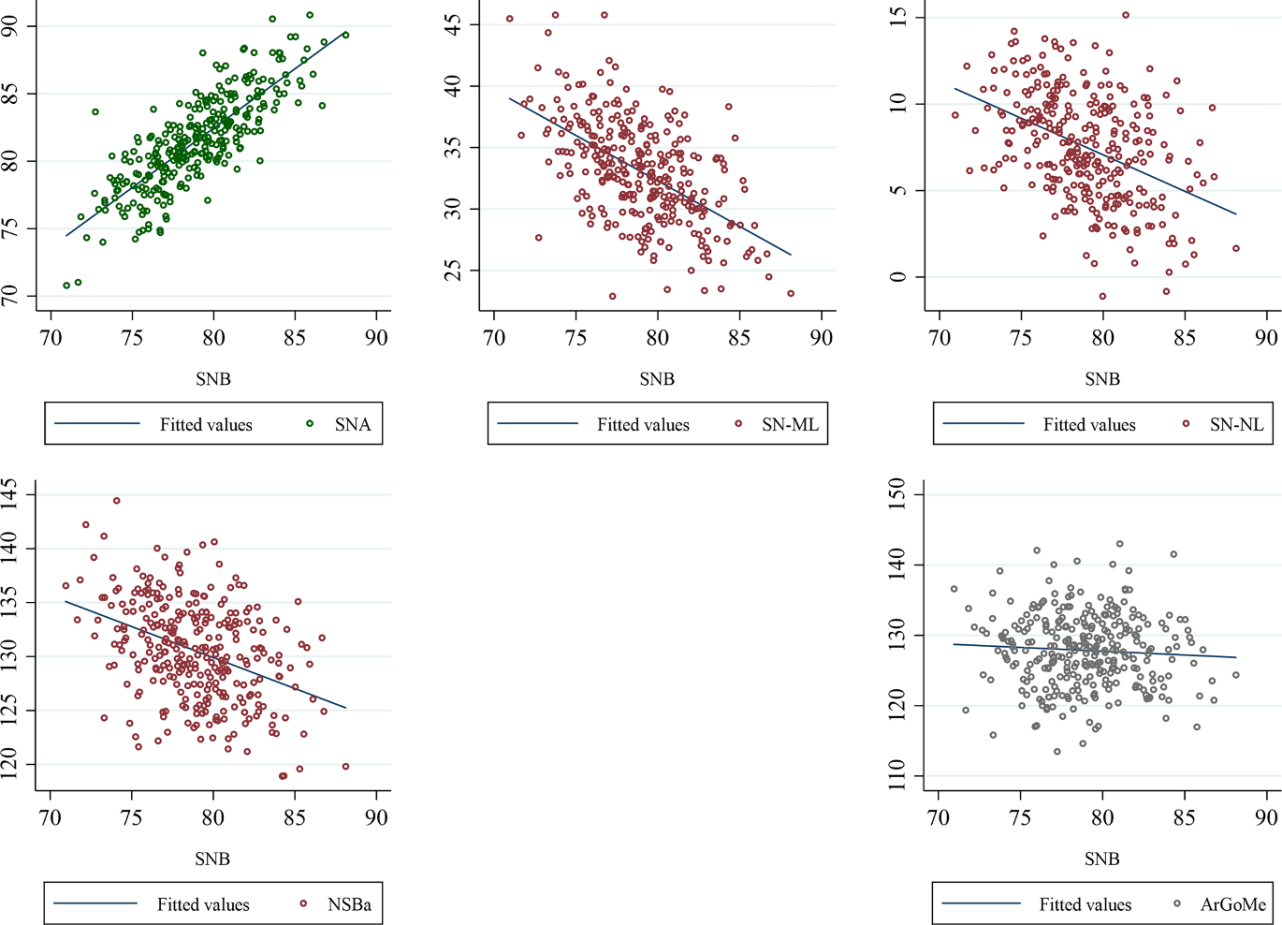
Scatterplots with fitted regression lines between the cephalometric variables framing the newly provided harmony box. The slope of the line indicates the level and direction of the correlation Streudiagramme mit angepassten Regressionsgeraden zwischen den kephalometrischen Variablen, welche die neu erstellte Harmoniebox eingrenzen. Die Steigung der Geraden gibt Niveau und Richtung der Korrelation an

**Fig. 2:**
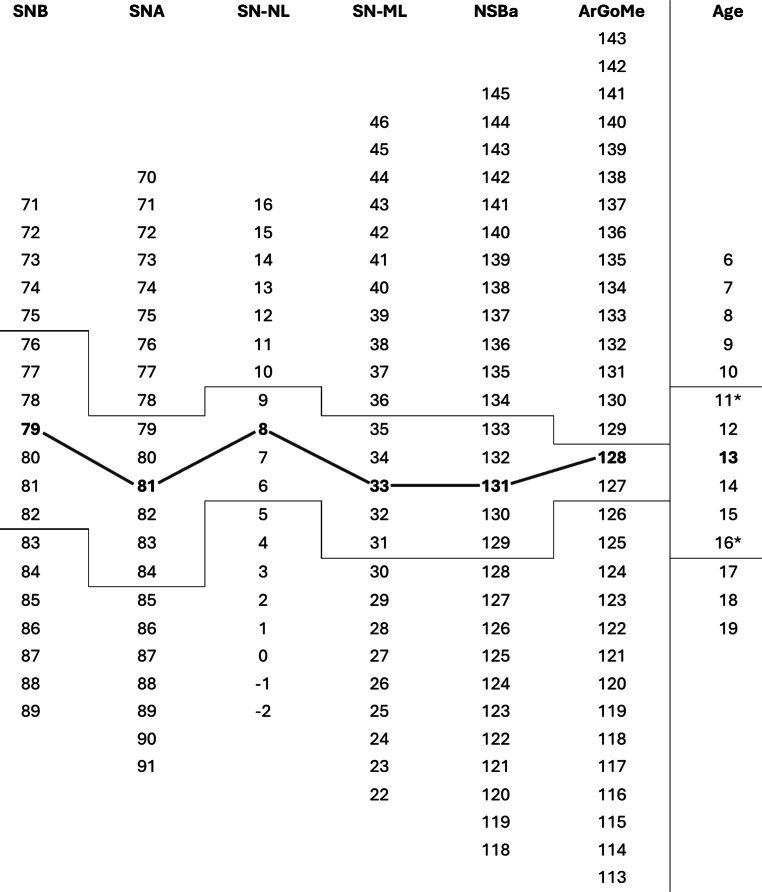
Values of the average patient outlined in the newly provided harmony box (values in bold and connecting lines). Also shown are lines representing values within (±) one standard deviation (SD). Asterisks represent age within the SD limits Werte des Durchschnittspatienten, angegeben in der neu erstellten Harmoniebox (Werte halbfett gesetzt, Verbindungslinien). Außerdem sind Linien dargestellt, die Werte innerhalb (±) einer Standardabweichung (SD) darstellen. Sternchen stehen für das Alter innerhalb der SD-Grenzen

The univariable regression functions based on the most stable model between variables that frame the newly provided harmony box are presented in Table 4. Furthermore, scatterplots with fitted regression lines between the cephalometric variables framing the new harmony box are also presented in Fig. 1. A graphical example is presented in Fig. 2.

**Table 4:** Univariable regression functions between variables that frame the newly provided harmony box^a^ Univariable Regressionsfunktionen zwischen Variablen, welche die neu bereitgestellte Harmoniebox^a^ umrahmen

y = ax + b^b^	SE	95%CI	R^2^
SNA = 0.88 × SNB + 12.00	0.04	0.81, 0.95	0.65
SN-ML = −0.74 × SNB + 91.52	0.06	−0.87, −0.61	0.31
SN-NL = −0.42 × SNB + 40.87	0.05	−0.52, −0.32	0.18
NSBa = −0.57 × SNB + 175.83	0.08	−0.73, −0.42	0.15
ArGoMe = −0.11 × SNB + 136.35	0.10	−0.30, 0.08	0.004
SNB = 0.27 × age + 75	0.07	0.13, 0.41	0.05

*y* dependent variable, *x* independent variable*, a* regression coefficient indicating change in y for each unit change in x, *b* constant indicating value of y when x = 0, *SE* standard error*, CI* confidence interval, *R*^*2*^ coefficient of determination

^a^ based on the most stable model as presented in Table 3

^b^ this does not imply plausible interpretation per se but contributes to the overall regression equation calculations

## Discussion

The present study assessed whether the gonial and interincisal angles qualify for inclusion in a new harmony box for individualized cephalometrics in a sample of Swiss nonadult patients. Based on the present results, the interincisal angle did not qualify for inclusion, yet the gonial angle was included in the new harmony box. The strongest correlations were observed between the cephalometric variables SNB and SNA, ArGoMe and SN-ML, and SN-ML and SNB. Furthermore, since the present study was conducted with material from the Zurich Craniofacial Growth Study, a wide range of ages from 6 to 18 years old was considered. In line with existing evidence [29–31], the SNB angle was found to be associated with age—the SNB angle increased with increasing age.

Segner [13] originally reported the correlation coefficients for the cephalometric variables included in the harmony box based on his sample of ideal occlusions. The present correlation coefficients for the cephalometric variables of the harmony box are comparable to those originally reported by Segner [13], which in turn were also similar to those reported by Järvinen [32] and Solow [11]. Compared to the results presented by Segner [13], the largest absolute difference of 0.12 in the correlation coefficients concerns the cephalometric pair NSBa and SN-ML, while the smallest absolute differences of 0.01 were between SN-ML and SN-NL, SNA and SNB, and SN-NL and SNB. All other correlation coefficients between the rest of cephalometric pairs did not present absolute differences greater than 0.07 [13]. Similarly, for the cephalometric characteristics of the sample, the average cephalometric values for the SNA, SN-NL, and NSBa angles did not differ more than 1° from Segner’s [13] sample. The largest average difference was recorded for the SN-ML angle with almost 5°, while the average difference for the SNB angle was approximately 1.6°. Compared to norms for the German and other populations [5, 33, 34], the average SN-ML angle of 28.2°, as reported by Segner [13] would seem rather small, presumably contributing to the difference in correlation coefficients regarding the cephalometric pair NSBa and SN-ML between Segner’s [13] sample and the present sample.

Additionally, compared with the Segner’s [13] sample, the present one appears slightly more retrognathic. This could possibly relate to the inclusion criteria, since Segner [13] included cases with ideal occlusions, whereas a range of cases with overjet and overbite from 2–4 mm and 1.5–4.5 mm, respectively, were included in the present study. One could further speculate that age differences could account for skeletal differences especially regarding the mandible, since we included growing patients, whereas the study of Segner [13] was conducted on young adults. As far as the overjet in the present study is concerned, this could be seen as a widely acceptable normal range [35]. However, the range of overbite might be considered slightly larger than the typically acceptable one. First, both overjet and overbite do not remain stable with increasing age [28, 36]. Since overbite changes more than overjet with age and deep overbites demonstrate a greater tendency to open than normal overbites, the present overbite cut-off values could be well justified [28]. Furthermore, normal dental and skeletal relationships are more about a wide continuum of values rather than specific cut-off values, even if the former is considered as deviating from the norm [37, 38]. Finally, the fact that occlusion at the molars was not considered due to missing data might be seen as a limitation in the present study. It seems, however, that overjet and overbite might be at least equally important as molar occlusion, since they define treatment need in well-established indices [39, 40].

Relative to the skeletal traits, it is widely believed that they are under a strong genetic influence [41]. Some authors claim that the vertical skeletal traits are more highly genetically determined than the sagittal skeletal ones [42, 43]. The interplay between the vertical and sagittal planes is a well-known orthodontic concept though [44]. On the other hand, intra-arch traits, such as arch form and crowding, do seem to demonstrate heritability as opposed to occlusal traits, such as overjet, overbite, and sagittal molar relationship, which do not seem to demonstrate heritability [45]. The dentoalveolar compensatory mechanism [46], by which the teeth and dental arches are secondarily adapted to the varying skeletal relationships, appears to further support the findings on the lack of heritability regarding occlusal traits. Based on the aforementioned, the failure of the interincisal angle to contribute to the stability and robustness of the multivariate model might be explained. Moreover, the qualification of the SNB angle as the outcome variable might depict the strong genetic control over the shape and sagittal position of the mandible [47]. Finally, following the addition of the ArGoMe angle, the new harmony box better approximates the polygon of the facial profile similarity, which refers to cephalometric angles with high heritability estimates and further includes the angles NSAr and SNB [47].

The concept of floating norms considering correlations between cephalometric variables for a harmonious appearance was the basis of the Segner–Hasund harmony box [13–15]. Here, the same concept was used to create a new harmony box with floating norms for a different population, i.e., growing Swiss individuals, as well as with the addition of the gonial angle and age. We used an array of statistical approaches to document all pairwise and multivariable relationships across the cephalometric variables, including both the ones defining the original harmony box, as well as the qualifying additional variables given also the characteristics of our sample (i.e., growing patients). The SNB angle served as the outcome variable, based on the stability of the models constructed, which adds value to the interpretation of our findings. This is considered a strength of the present work, which resulted in the addition of two variables to better describe the specifics of the present Swiss sample. On the other hand, since the foundation of the newly provided harmony box lies on the predefined cephalometric variables of the original Segner–Hasund harmony box [13–15], one could potentially argue that important intercorrelations between similar and/or additional cephalometric variables better applying to the present sample might have been ignored.

The findings of this study may be generalizable to similar populations, following the characteristics of our sample. However, one should bear in mind that the selection of different outcome variables and different models including predictor variables, might produce possibly variable harmony boxes, and apply to more heterogeneous populations [48].

## Conclusions

Based on the original harmony box for individualized cephalometrics, a new harmony box was created with the addition of the gonial angle as well as age for a sample of healthy schoolchildren with acceptable overjet and overbite from Zurich, Switzerland.

## Supplementary Information

**Appendix 1: **Angular cephalometric measurements used in this study along with their definitions. **Appendix 2: **Concordance and agreement of intra- and interexaminer angular cephalometric measurements

## Data Availability

Data will be made available on reasonable request.

## References

[1] Broadbent BH (1931) A new x‑ray technique and its application to orthodontia. Angle Orthod 1:45–66. https://doi.org/10.1043/0003-3219(1931)001〈0045:ANXTAI〉2.0.CO;2

[2] Hofrath H (1931) Die Bedeutung der Röntgenfern- und Abstandsaufnahme für die Diagnostik der Kieferanomalien. Fortschritte Orthod 1:232–258. 10.1007/BF02002578

[3] Nielsen IL (2022) Cephalometric analysis: history and clinical application. Taiwan J Orthod 34:175–184. 10.38209/2708-2636.1323

[4] Keim RG, Gottlieb EL, Nelson AH, Vogels DS (2008) 2008 JCO study of orthodontic diagnosis and treatment procedures, part 1: results and trends. J Clin Orthod 42:625–640 19075377

[5] Athanasiou AE (1995) Orthodontic cephalometry. Mosby-Wolfe, London

[6] Graber TM (1956) Problems and limitations of cephalometric analysis in orthodontics. J Am Dent Assoc 53:439–454. 10.14219/jada.archive.1956.0204 13366566 10.14219/jada.archive.1956.0204

[7] Hixon EH (1956) The norm concept and cephalometrics. Am J Orthod 42:898–906. 10.1016/0002-9416(56)90190-7

[8] Bonnefont R, Ernoult J‑F, Sorel O (2011) A new method of using cephalometric measurements in orthodontics (part 2) or how standard deviations can be the practitioner’s false friends. J Dentofacial Anom Orthod 14:1–18. 10.1051/odfen/2011104

[9] McDermid R (2021) Statistics in medicine. Anaesth Intensive Care Med 22:454–462. 10.1016/j.mpaic.2021.05.009

[10] Enlow DH, Hans MG (1996) Essentials of facial growth. Saunders, Philadelphia

[11] Solow B (1966) The pattern of craniofacial associations. A morphological and methodological correlation and factor analysis study on young male adults. Acta Odontol Scand 24: (Supplementum)

[12] Hasund A, Böe O, Jenatsche F et al (1974) Clinical Cephalometry for the Bergen technique. University of Bergen, Bergen

[13] Segner D (1989) Floating norms as a means to describe individual skeletal patterns. Eur J Orthod 11:214–220. 10.1093/oxfordjournals.ejo.a035988 2676569 10.1093/oxfordjournals.ejo.a035988

[14] Segner D, Hasund A (1994) Individualisierte Kephalometrie, 2nd edn. Dietmar Segner Verl, Hamburg

[15] Beckmann SH, Segner D (2002) Floating norms and post-treatment overbite in open bite patients. Eur J Orthod 24:379–390. 10.1093/ejo/24.4.379 12198868 10.1093/ejo/24.4.379

[16] Björk A (1947) The Face in Profile. An anthropological X‑ray investigation on Swedish children and conscripts. Berlingska Boktryckeriet, Lund

[17] Björk A (1969) Prediction of mandibular growth rotation. Am J Orthod 55:585–599. 10.1016/0002-9416(69)90036-0 5253957 10.1016/0002-9416(69)90036-0

[18] Sassouni V (1969) A classification of skeletal facial types. Am J Orthod 55:109–123. 10.1016/0002-9416(69)90122-5 5249177 10.1016/0002-9416(69)90122-5

[19] D’Antò V, Pango Madariaga AC, Rongo R et al (2019) Distribution of the Condylion-Gonion-Menton (cogome^) angle in a population of patients from southern Italy. Dent J 7:104. 10.3390/dj7040104 10.3390/dj7040104 PMC6960630 31684195

[20] Krüsi M, Halazonetis DJ, Eliades T, Koretsi V (2023) Covariance patterns between ramus morphology and the rest of the face: a geometric morphometric study. Korean J Orthod 53:185–193. 10.4041/kjod22.208 37113038 10.4041/kjod22.208 PMC10212770

[21] Carpiaux W (2014) Geometric morphometrics clusters craniofacial morphology differently than traditional cephalometric measurements. Temple University (M.S.)

[22] Halazonetis DJ (2004) Morphometrics for cephalometric diagnosis. Am J Orthod Dentofacial Orthop 125:571–581. 10.1016/j.ajodo.2003.05.013 15127026 10.1016/j.ajodo.2003.05.013

[23] Alhammadi M‑S (2019) Dentoalveolar compensation in different anterioposterior and vertical skeletal malocclusions. J Clin Exp Dent 11:e745–e753. 10.4317/jced.56020 31598204 10.4317/jced.56020 PMC6776409

[24] Molina-Berlanga N, Llopis-Perez J, Flores-Mir C, Puigdollers A (2013) Lower incisor dentoalveolar compensation and symphysis dimensions among Class I and III malocclusion patients with different facial vertical skeletal patterns. Angle Orthod 83:948–955. 10.2319/011913-48.1 23758599 10.2319/011913-48.1 PMC8722832

[25] Kim H‑J, Noh H‑K, Park H‑S (2023) Mandibular asymmetry types and differences in dental compensations of Class III patients analyzed with cone-beam computed tomography. Angle Orthod 93:695–705. 10.2319/013023-73.1 37407513 10.2319/013023-73.1 PMC10633797

[26] Sarver DM (2001) The importance of incisor positioning in the esthetic smile: the smile arc. Am J Orthod Dentofacial Orthop 120:98–111. 10.1067/mod.2001.114301 11500650 10.1067/mod.2001.114301

[27] Lorenz N, Patcas R, Alexopoulou E et al (2022) Individualized cephalometry: an update of the Segner-Hasund harmony box. https://osf.io/nvkt4/. Accessed 3 June 2024

[28] Björk A (1953) Variability and age changes in overjet and overbite: report from a follow-up study of individuals from 12 to 20 years of age. Am J Orthod 39:779–801. 10.1016/0002-9416(53)90084-0

[29] van Diepenbeek AF, Buschang PH, Prahl-Andersen B (2009) Age-dependant cephalometric standards as determined by multilevel modeling. Am J Orthod Dentofacial Orthop 135:79–87. 10.1016/j.ajodo.2006.11.025 19121505 10.1016/j.ajodo.2006.11.025

[30] Bishara SE (1981) Longitudinal cephalometric standards from 5 years of age to adulthood. Am J Orthod 79:35–44. 10.1016/0002-9416(81)90099-3 6935970 10.1016/0002-9416(81)90099-3

[31] Nanda RS, Ghosh J (1995) Longitudinal growth changes in the sagittal relationship of maxilla and mandible. Am J Orthod Dentofacial Orthop 107:79–90. 10.1016/S0889-5406(95)70159-1 7817964 10.1016/s0889-5406(95)70159-1

[32] Järvinen S (1986) Floating norms for the ANB angle as guidance for clinical considerations. Am J Orthod Dentofacial Orthop 90:383–387. 10.1016/0889-5406(86)90004-1 3465233 10.1016/0889-5406(86)90004-1

[33] Reich U, Dannhauer K‑H (1996) Craniofacial morphology of orthodontically untreated patients living in Saxony, Germany. J Orofac Orthop Fortschr Kieferorthop 57:246–258. 10.1007/BF02190237 10.1007/BF02190237 8765800

[34] Stahl de Castrillon F, Baccetti T, Franchi L et al (2013) Lateral cephalometric standards of Germans with normal occlusion from 6 to 17 years of age. J Orofac Orthop 74:236–256. 10.1007/s00056-013-0140-5 23649277 10.1007/s00056-013-0140-5

[35] Kinaan BK (1986) Overjet and overbite distribution and correlation: a comparative epidemiological English-Iraqi study. Br J Orthod 13:79–86. 10.1179/bjo.13.2.79 3456795 10.1179/bjo.13.2.79

[36] Ceylan I, Baydas B, Bölükbasi B (2002) Longitudinal cephalometric changes in incisor position, overjet, and overbite between 10 and 14 years of Age. Angle Orthod 72:246–250. https://doi.org/10.1043/0003-3219(2002)072〈0246:LCCIIP〉2.0.CO;2 12071608 10.1043/0003-3219(2002)072<0246:LCCIIP>2.0.CO;2

[37] Kim J‑Y, Lee S‑J, Kim T‑W et al (2005) Classification of the skeletal variation in normal occlusion. Angle Orthod 75:311–319. 10.1043/0003-3219(2005)75%5B311:COTSVI%5D2.0.CO;2 15898366 10.1043/0003-3219(2005)75[311:COTSVI]2.0.CO;2

[38] Ku S‑J, Lee S‑J, Chang Y‑I (2002) Dentoalveolar compensation according to skeletal patterns of normal occlusion. Korean J Orthod 32:91–105

[39] Burden DJ, Pine CM, Burnside G (2001) Modified IOTN: an orthodontic treatment need index for use in oral health surveys. Community Dent Oral Epidemiol 29:220–225. 10.1034/j.1600-0528.2001.290308.x 11409681 10.1034/j.1600-0528.2001.290308.x

[40] Gemeinsamer Bundesausschuss Anlage 1: Kieferorthopädische Indikationsgruppen. https://www.g-ba.de/richtlinien/anlage/54/. Accessed 9 Sept 2024

[41] Manfredi C, Martina R, Grossi GB, Giuliani M (1997) Heritability of 39 orthodontic cephalometric parameters on MZ, DZ twins and MN-paired singletons. Am J Orthod Dentofacial Orthop 111:44–51. 10.1016/S0889-5406(97)70301-9 9009923 10.1016/s0889-5406(97)70301-9

[42] Hunter WS (1965) A study of the inheritance of craniofacial characteristics as seen in lateral cephalograms of 72 like-sexed twins. Rep Congr Eur Orthod Soc 41:59–70 5222375

[43] Lundström A, McWilliam JS (1987) A comparison of vertical and horizontal cephalometric variables with regard to heritability. Eur J Orthod 9:104–108. 10.1093/ejo/9.2.104 3472887 10.1093/ejo/9.2.104

[44] Hussels W, Nanda RS (1984) Analysis of factors affecting angle ANB. Am J Orthod 85:411–423. 10.1016/0002-9416(84)90162-3 6586080 10.1016/0002-9416(84)90162-3

[45] Santana LG, Flores-Mir C, Iglesias-Linares A et al (2020) Influence of heritability on occlusal traits: a systematic review of studies in twins. Prog Orthod 21:29. 10.1186/s40510-020-00330-8 32864724 10.1186/s40510-020-00330-8 PMC7456624

[46] Solow B (1980) The Dentoalveolar compensatory mechanism: background and clinical implications. Br J Orthod 7:145–161. 10.1179/bjo.7.3.145 6934010 10.1179/bjo.7.3.145

[47] Šidlauskas M, Šalomskienė L, Andriuškevičiūtė I et al (2016) Heritability of mandibular cephalometric variables in twins with completed craniofacial growth. Eur J Orthod 38:493–502. 10.1093/ejo/cjv062 26503948 10.1093/ejo/cjv062

[48] Bingmer M, Özkan V, Jo J et al (2010) A new concept for the cephalometric evaluation of craniofacial patterns (multiharmony). Eur J Orthod 32:645–654. 10.1093/ejo/cjp152 20305056 10.1093/ejo/cjp152

