# Supplementary material for: A new harmony box supplemented with gonial angle and age based on a growing Swiss population
Source: J Orofac Orthop. 2025 Jan 15;86(Suppl 1):111–9. doi: 10.1007/s00056-024-00569-4 (PMC12394316; doi:10.1007/s00056-024-00569-4)
Supplement: Supplementary file 1 — Appendix 1: Angular cephalometric measurements used in this study along with their definitions. Appendix 2: Concordance and agreement of intra- and interexaminer angular cephalometric measurements [file 56_2024_569_MOESM1_ESM.pdf]

Appendix

Appendix 1 Angular cephalometric measurements used in this study along with their definitions

Variable	Definition
ANB	The angle formed by the landmarks A point, B point, and Nasion
SNA	The angle formed by the anterior cranial base (Sella to Nasion) and landmark A point
SN-NL	The angle formed by the anterior cranial base (Sella to Nasion) and the maxillary plane (anterior nasal spine to posterior nasal spine)
NSBa	The angle formed by the anterior cranial base (Sella to Nasion) and Basion
SN-ML	The angle formed by the anterior cranial base (Sella to Nasion) and the mandibular plane (Gonion to Menton)
SNB	The angle formed by the anterior cranial base (Sella to Nasion) and landmark B point
NL-ML	The angle formed by the maxillary plane (anterior nasal spine to posterior nasal spine) and the mandibular plane (Gonion to Menton)
U1-L1	The angle formed by the long axes of the maxillary and mandibular incisors
ArGoMe	The gonial angle, formed by the landmarks Articulare, Gonion, and Menton

Appendix 2 Concordance and agreement of intra- and inter-examiner angular cephalometric measurements

Variable	Assessment	Lin's CCC (95%CI)	Difference (95% LoA)	p-value[a]
	**Intra-examiner**			
**NSBa**		0.984 (0.975, 0.993)	0.100 (-1.389, 1.588)	0.85
**SNA**		0.993 (0.989, 0.997)	0.054 (-0.843, 0.952)	0.27
**SNB**		0.993 (0.990, 0.997)	0.076 (-0.780, 0.931)	0.134
**ANB**		0.979 (0.967, 0.991)	-0.022 (-0.920, 0.877)	0.68
**SN-ML**		0.983 (0.974, 0.993)	-0.080 (-1.942, 1.781)	0.39
**SN-NL**		0.991 (0.987, 0.996)	-0.090 (-0.958, 0.777)	0.06
**ML-NL**		0.989 (0.983, 0.995)	0.010 (-1.603, 1.624)	0.92
**ArGoMe**		0.990 (0.985, 0.996)	0.008 (-1.499, 1.515)	0.84
**U1-L1**		0.994 (0.991, 0.997)	0.088 (-1.525, 1.700)	0.09
	**Inter-examiner**			
**NSBa**		0.987 (0.980, 0.994)	-0.281 (-1.513, 0.951)	0.94
**SNA**		0.990 (0.985, 0.996)	0.230 (-0.706, 1.165)	0.94
**SNB**		0.992 (0.988, 0.997)	0.230 (-0.580, 1.040)	0.54
**ANB**		0.973 (0.958, 0.988)	-0.001 (-1.026, 1.024)	0.35
**SN-ML**		0.983 (0.974, 0.992)	-0.394 (-2.118, 1.331)	0.12
**SN-NL**		0.988 (0.981, 0.995)	-0.222 (-1.166, 0.722)	0.34
**ML-NL**		0.990 (0.984, 0.995)	-0.173 (-1.728, 1.382)	0.27
**ArGoMe**		0.991 (0.986, 0.996)	-0.074 (-1.529, 1.381)	0.67
**U1-L1**		0.995 (0.992, 0.998)	0.296 (-1.063, 1.656)	0.79

CCC concordance correlation coefficient, *CI* confidence interval, *LoA* limits of agreement

[a] Pitman's test of difference in variance
